# Identification of cell subpopulations associated with disease phenotypes from scRNA-seq data using PACSI

**DOI:** 10.1186/s12915-023-01658-3

**Published:** 2023-07-19

**Authors:** Chonghui Liu, Yan Zhang, Xin Gao, Guohua Wang

**Affiliations:** 1 College of Life Science, Northeast Forestry University, Harbin, 150040 China; 2 College of Computer and Control Engineering, Northeast Forestry University, Harbin, 150040 China; 3 Department of Ophthalmology, the Second Affiliated Hospital of Harbin Medical University, Harbin, 150086 China; 4 Computer Science Program, Computer, Electrical and Mathematical Sciences and Engineering (CEMSE) Division, King Abdullah University of Science and Technology (KAUST), Thuwal, 23955-6900 Kingdom of Saudi Arabia; 5 KAUST Computational Bioscience Research Center (CBRC), King Abdullah University of Science and Technology, Thuwal, 23955-6900 Kingdom of Saudi Arabia; 6 School of Computer Science and Technology, Harbin Institute of Technology, Harbin, 150001 China

**Keywords:** Single-cell, PPI, Phenotype, Cancer, COVID-19, Immunotherapy, Spatial transcriptomics

## Abstract

**Background:**

Single-cell RNA sequencing (scRNA-seq) has revolutionized the transcriptomics field by advancing analyses from tissue-level to cell-level resolution. Despite the great advances in the development of computational methods for various steps of scRNA-seq analyses, one major bottleneck of the existing technologies remains in identifying the molecular relationship between disease phenotype and cell subpopulations, where “disease phenotype” refers to the clinical characteristics of each patient sample, and subpopulation refer to groups of single cells, which often do not correspond to clusters identified by standard single-cell clustering analysis. Here, we present PACSI, a method aimed at distinguishing cell subpopulations associated with disease phenotypes at the single-cell level.

**Results:**

PACSI takes advantage of the topological properties of biological networks to introduce a proximity-based measure that quantifies the correlation between each cell and the disease phenotype of interest. Applied to simulated data and four case studies, PACSI accurately identified cells associated with disease phenotypes such as diagnosis, prognosis, and response to immunotherapy. In addition, we demonstrated that PACSI can also be applied to spatial transcriptomics data and successfully label spots that are associated with poor survival of breast carcinoma.

**Conclusions:**

PACSI is an efficient method to identify cell subpopulations associated with disease phenotypes. Our research shows that it has a broad range of applications in revealing mechanistic and clinical insights of diseases.

**Supplementary Information:**

The online version contains supplementary material available at 10.1186/s12915-023-01658-3.

## Background

Single-cell RNA sequencing (scRNA-seq) is revolutionizing whole-transcriptomic studies from the tissue resolution to the cell resolution [1]. Despite the great advances in the development of computational methods for various steps of scRNA-seq analyses, one major bottleneck of the existing technologies is identifying the molecular relationship between disease phenotype and cell populations. We use “disease phenotypes” as the clinical characteristics of each patient sample, such as disease vs. normal, poor survival vs. good survival, responder vs. non-responder, and so on [2]. Disease phenotypes of interest are frequently driven by some critical cells with abnormal function or activity [3–6]. Recognizing the cell subpopulations associated with disease phenotype from single-cell data is of fundamental importance because it will assist in cell population-specific targeted therapies and the discovery of biological biomarkers [7, 8].

There are multiple statistical methods that have been developed to explore single-cell data. Seurat utilizes unsupervised clustering to identify cell types and then associate cell types with disease phenotypes [9]. However, many scRNA-seq studies include only a small number of patient samples and generate a lot of cells for each patient sample [10], making this strategy less statistically powerful. As alternative strategies, deconvolution [11, 12] and single-sample gene set enrichment analysis (ssGSEA) [13] have also been used to identify cell subsets associated with disease phenotypes. These methods assess the association of disease phenotypes with the previously defined cell clusters rather than individual cells. In other words, these methods fail to distinguish cells associated with disease phenotype from single-cell data, especially if the target cells are distributed in diverse cell clusters. Moreover, they only compare the abundance of cell types between samples, neglecting transcriptional changes of these cells.

To address these challenges, Scissor was purposed to dissect phenotype-specific cell subsets from heterogeneous single-cell data [14]. The key step of Scissor is employing Pearson correlation at the whole transcriptome level to quantify the similarity between cells and samples. Although this method focuses on the importance of genetic perturbations of cells, such a whole-transcriptome perspective may overlook changes in gene expression of a small number of key genes. Currently, DEGAS combined deep learning and transfer learning to transfer phenotype information from patients to cells [15]. The main drawback of this strategy is the lack of effective biological interpretation. In summary, there is an urgent need for a method with both superior performance and good interpretability to identify cell subpopulations associated with disease phenotypes from single-cell data.

Therefore, we have developed PACSI (Phenotype-Associated Cell Subpopulation Identification), a novel network-based method to identify cell subpopulations associated with disease phenotypes of interest. PACSI takes a single-cell transcriptome dataset, a bulk gene expression matrix, phenotype labels and protein-protein interaction (PPI) networks as inputs. PACSI consists of three steps: (1) cell/sample signatures in the form of gene sets are constructed using the highly expressed genes of a cell/sample relative to the others in the single-cell or bulk gene expression matrix; (2) network-based proximity is calculated to define similarity between cells and the disease phenotype of interest; (3) the significance of the proximity-based similarity between a cell and the phenotype of interest is assessed by randomly assigning genes in the cell signature. We tested PACSI on multiple datasets of various disease phenotypes to ascertain the broad utilities of PACSI. Our studies suggest that PACSI allows scientists to generate more biological insights into the underlying mechanisms of complex diseases, which can promote the development of precision medicine.

## Results

### PACSI: a graph-based approach for identifying cell subpopulations associated with disease phenotypes

**Fig. 1:**
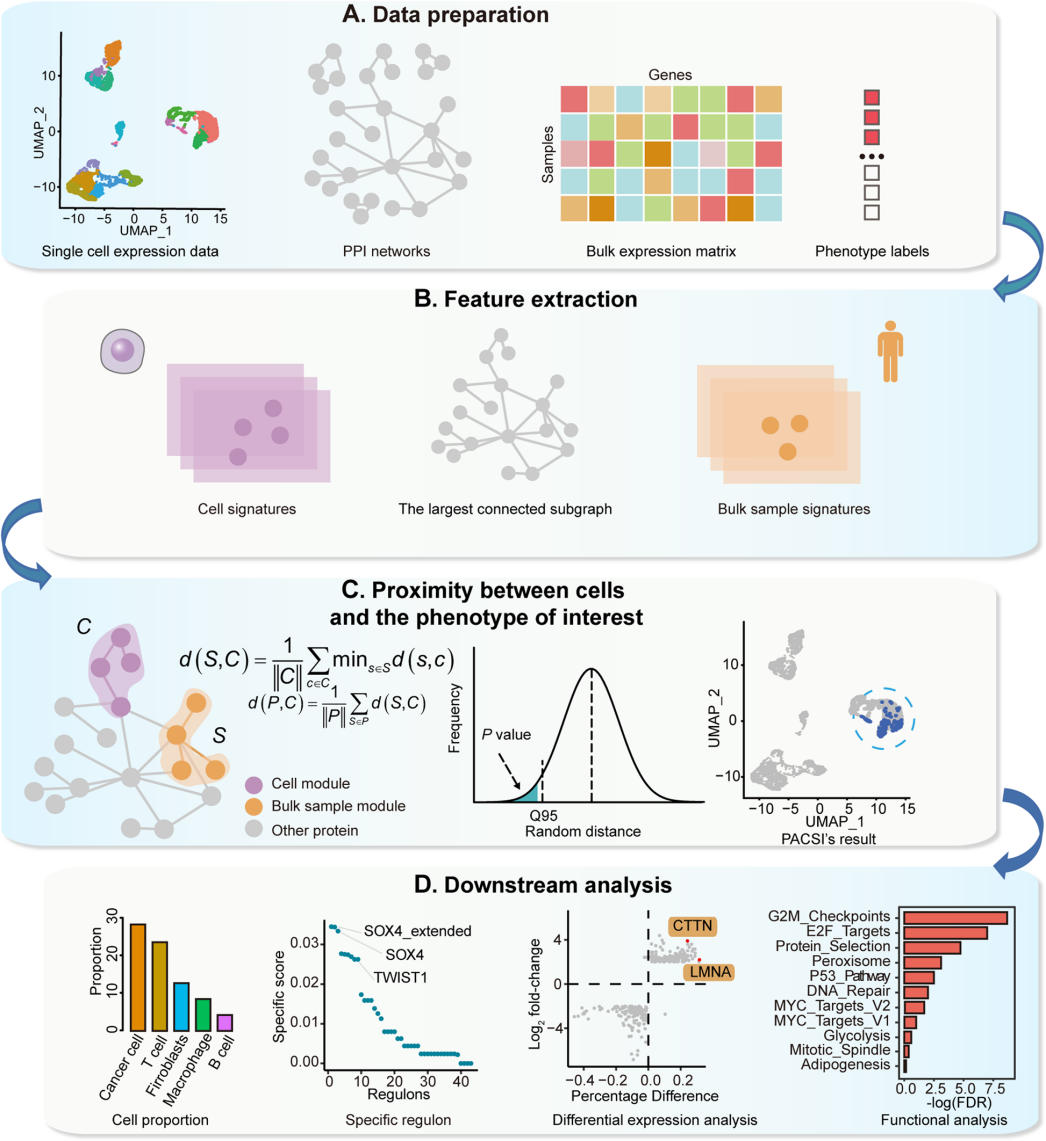
The workflow diagram of PACSI. **A** The inputs for PACSI are the scRNA-seq data, the bulk expression data, phenotype labels corresponding to bulk data and PPI networks. **B** PACSI extracts separately the cell signatures and bulk sample signatures from scRNA-seq data and bulk expression data, respectively (left and right). Meanwhile, PACSI calculated the largest connected component of the PPI network using the R igraph package (middle). **C** Each cell signature and each bulk sample signature respectively induces a module in the largest connected component of the PPI network, and then the network-based cell-phenotype proximity is calculated (left). Next, the PACSI results are evaluated by calculating empirical *P* values from random permutations (middle). The PACSI-identified cells can be visualized using UMAP (right). **D** The cell subpopulations identified by PACSI are used for downstream analysis

To develop a general-purpose algorithm that is suitable for many disease phenotypes, we integrated single-cell expression matrices, bulk gene expression data, bulk sample phenotype labels, and PPI networks to identify cells related to disease phenotypes (Fig. 1A). The first step of PACSI was to obtain a gene signature for each cell and each bulk sample. PACSI first selected highly expressed genes as the gene expression signature for each cell and bulk sample (Fig. 1B, left and right). In addition, PACSI extracted the largest connected component of the PPI network to calculate the network distance between each cell and each bulk sample in subsequent analysis (Fig. 1B, middle). After this, each cell or bulk sample signature was genetically characterized and induced a module in the largest connected component of the PPI network (Fig. 1C, left). For each cell-sample pair, we computed the average shortest path length between each cell module and bulk sample module to quantify the correlation for each cell-sample pair. To obtain the final network-based relationship between cells and the phenotype of interest, we averaged the shortest paths between each cell and the bulk samples with the phenotype labels of interest. Furthermore, PACSI created randomly a reference distance distribution to assess the significance of the relationship between cells and the phenotype of interest (Fig. 1C, middle). Finally, the utility of PACSI-selected cells (Fig. 1C, right) was illustrated in downstream analyses, such as specific regulatory analysis and functional analysis (Fig. 1D).

### PACSI correctly detected the phenotype-related subpopulations in simulated data

**Fig. 2:**
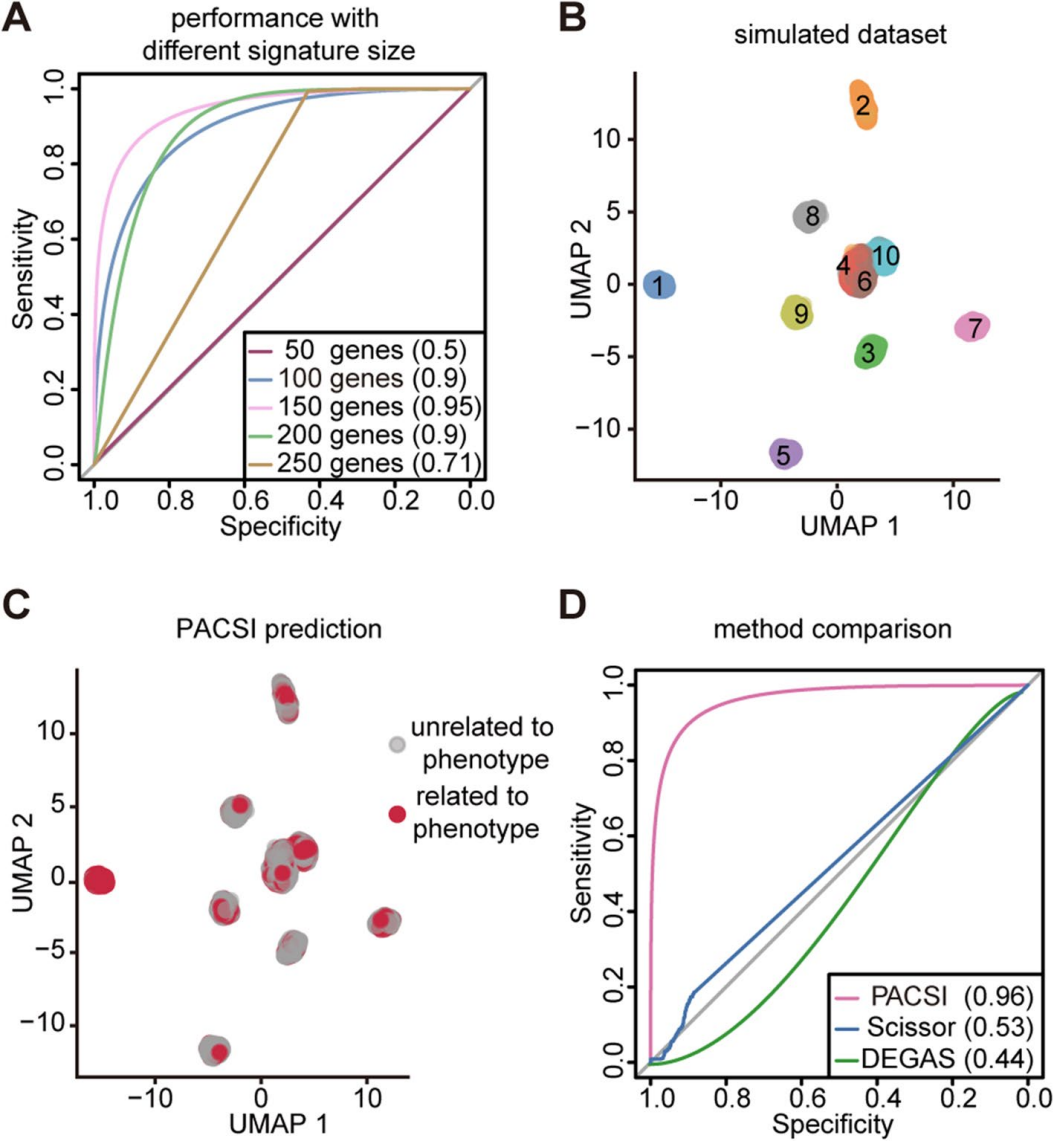
Simulation data shows the advantages of PACSI. **A** The ROC curves of PACSI with various sizes of cell/sample signatures. **B** The UMAP visualization of simulated single-cell data. **C** The UMAP visualization of PACSI-identified cells. The red dots are PACSI-identified cells associated with phenotype and the gray dots represent the rest of the cells in the simulated single-cell data. **D** The ROC curves of three methods

To evaluate the performance of PACSI in a controlled context, we implemented splatter [16] to generate single-cell and bulk RNA-sequencing FPKM data. We first simulated 5000 cells, forming 10 groups of the same size with 10,000 genes per cell (Additional file 1: Supplementary Fig. 1A), and 500 samples. We defined the first cluster of cells as the ground truth. We employed the receiver operating characteristic (ROC)–area under the curve (AUC) and the precision-recall (PR)-area under the curve (AUPR) as measures of predictive performance due to the large imbalance of classes. We first evaluated how the accuracy of PACSI changes in regard to the size of cell/sample signatures. Figure 2A showed the performance of PACSI when the size of cell/sample signatures varies from 50 to 250, and we found that the size of signatures did influence the performance of PACSI. PACSI performed best with 150-gene signature in simulated data, so we set 150 as the default parameter. After that, to further assess the performance of PACSI, we regenerated a new single-cell expression profile (Fig. 2B) and bulk expression profile using the same method and parameters and compared PACSI with other methods reported previously, including Scissor and DEGAS. We have used these published methods with their default hyperparameters from provided tutorials. The results showed that PACSI achieved an AUC of 0.96 and an AUPR of 0.99 which were substantially higher than other methods for identifying the ground truth cells on the simulated dataset (Fig. 2C, D and Additional file 1: Supplementary Fig. 1B, C). Overall, the above results indicated that PACSI could be an effective method to accurately identify cell subpopulations associated with disease phenotypes.

### Capturing subpopulations related to HNSC

**Fig. 3:**
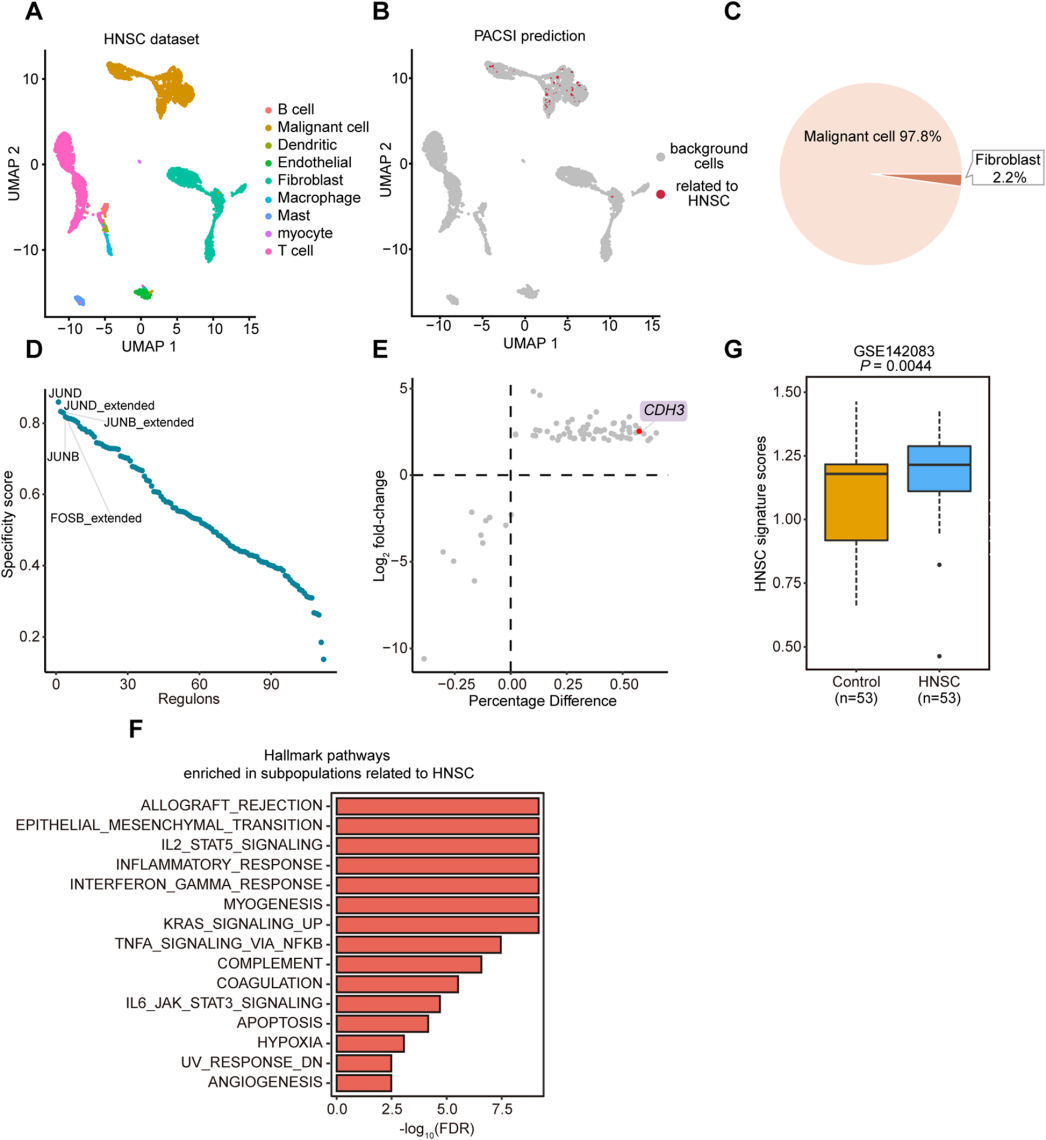
Evaluation of PACSI on HNSC data. **A** The UMAP visualization of the HNSC scRNA-seq dataset. **B** The UMAP visualization of PACSI-identified cells. The red dots are PACSI-identified cells associated with HNSC. **C** The distribution of PACSI-identified cells by cell types. **D** Rank for regulons in PACSI-identified cells associated with HNSC based on RSS. **E** Differential gene expression analysis. The *x*-axis shows the difference in the percentage of cells expressing the gene between PACSI-identified cells and the others; the *y*-axis represents the log_2_ fold-change. **F** The significantly enriched Hallmark pathways in the PACSI-identified cells compared to other cells using GSEA. **G** Box plot shows the enrichment scores of the HNSC signature in the HNSC and normal samples from the independent validation dataset. A two-sided Wilcoxon rank-sum test was performed to estimate the significance level

Head and neck squamous cell carcinoma (HNSC) are the most common malignant tumors that arise in the head and neck [17]. The identification of HNSC-related cells is critical to the biology, diagnosis, and treatment of HNSC. We first downloaded 69,567 experimentally verified human PPIs data from the MINT database and used this data for all real cases in this study [18]. The largest connected component extracted by PACSI retained more than 99% of the edges in the original PPI network. We employed PACSI, guided by 544 TCGA-HNSC bulk samples with the phenotype information, to infer cell subsets that were associated with HNSC within cells from the HNSC single-cell dataset. Among the 4244 cells from different cell types (Fig. 3A), 46 cells were identified by PACSI to be associated with tumor phenotype (Fig. 3B). Forty-five out of the 46 identified cells were malignant cells; the other one cell was fibroblast (Fig. 3C).

Consequently, to systematically infer crucial regulators for cell subpopulations identified by PACSI, we performed comprehensive gene regulatory network analysis (the “Methods” section). The regulators that were specific to the identified cells were arranged from large to small according to the regulon specificity score (RSS) [19]. The top three regulators were JUND, JUNB, and FOSB (Fig. 3D and Additional file 2). All three factors are well known oncogenes [20–23]. Thereafter, we compared gene expression of PACSI-identified cells with the others to detect transcriptional changes in these cells. As a result, 65 upregulated genes and 11 downregulated genes were uncovered to be differently expressed in these cells (Fig. 3E and Additional file 2). Multiple upregulated genes are related to HNSC such as *CDH3* [24] (Fig. 3E and Additional file 1: Supplementary Fig. 2). Gene set enrichment analysis using the Hallmark gene sets showed that the differentially expressed genes (DEGs) were significantly enriched in several pathways, such as epithelial-mesenchymal transition [25] and angiogenesis [26], which are closely related to HNSC (Fig. 3F and Additional file 2). Finally, to explore the clinical relevance of PACSI-derived signature (defined as the upregulated genes of the identified cells related to HNSC; Additional file 2), we performed ssGSEA on independent data. We found that the PACSI-derived signature scores were significantly higher in tumor samples than in normal tissues from HNSC, suggesting that the HNSC signature was indeed associated with HNSC and therapeutic strategies might be developed to target these genes (Fig. 3G).

### Identification of cell subpopulations associated with poor survival in breast carcinoma

**Fig. 4:**
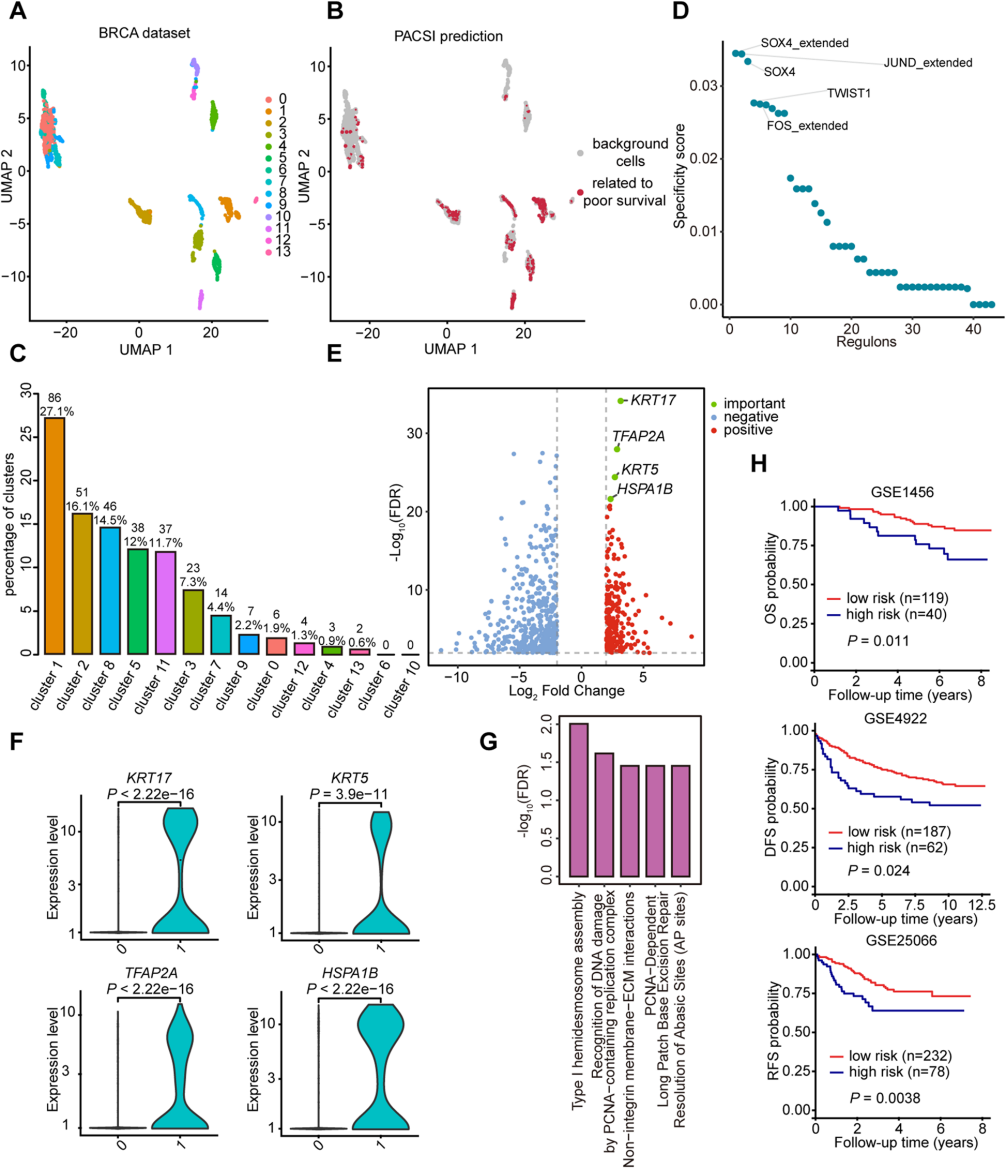
Application of PACSI on BRCA data. **A** The UMAP visualization of the BRCA scRNA-seq dataset. **B** The UMAP visualization of PACSI-identified cells. The red dots are PACSI-identified cells associated with poor prognosis in BRCA. **C** The distribution of PACSI-identified cells by cell clusters. **D** Rank for regulons in PACSI-identified cells associated with poor prognosis in BRCA based on RSS. **E** The differentially expressed genes (log_2_ fold change > 2, FDR < 0.01) in PACSI-identified cells (labeled 1) and all the other cells (labeled 0). **F** Violin plots show the expression levels of vital genes in PACSI-identified cells (*n* = 317) and all the other cells (*n* = 1217). Two-tailed *P* value was calculated by Wilcoxon rank-sum test. **G** The top five Reactome pathways enrichment of genes that were expressed higher in the PACSI-identified cells. **H** Kaplan–Meier estimates survival curves for the high-risk and low-risk groups according to PACSI-derived signature. The log-rank test was used to calculate *P* values. The *x*-axis indicates the follow-up time; the *y*-axis indicates the probability of overall survival (OS), disease-free survival (DFS), and recurrence-free survival (RFS)

Our study also extensively explored the ability of PACSI to identify cells related to poor survival. We applied PACSI on a single-cell dataset of 1534 cells from six breast cancer tumors [27] (Fig. 4A). The TCGA-BRCA bulk gene expression data and corresponding survival information were downloaded from the UCSC Xena database [28]. We identified a total of 317 cells related to poor survival in BRCA (Fig. 4B), among which clusters 1 and 2 were the two main cell types (Fig. 4C). For transcriptional regulatory analysis of PACSI-identified cells, SOX4, JUND, TWIST1, and FOS were identified as the most specific regulators (Fig. 4D and Additional file 3). Moreover, SOX4 can promote the growth and metastasis of breast carcinoma and has been proposed as a biomarker of poor prognosis in breast carcinoma patients [29, 30]. TWIST1 has also been shown to promote breast carcinoma invasion and metastasis [31], and high expression of TWIST1 has been found to be associated with poor prognosis in breast carcinoma [32].

We conducted a similar DEGs analysis comparing PACSI-identified cells versus the others. As shown in Fig. 4E, 692 transcripts were significantly differentially expressed in the identified cells (Additional file 3). Multiple upregulated genes in identified cells are associated with the prognosis of breast carcinoma patients (Fig. 4F). For example, it has been shown that *KRT17* and *KRT5* were significantly upregulated in basal-like breast carcinomas, and the overexpression of *KRT17* was associated with poor prognosis of cancer [33–35]. In addition, Ding et al. [36] reported that *TFAP2A* was aberrantly upregulated in breast carcinoma tissues and was associated with breast cancer progression. Besides, it has also been shown that high *HspA1B* expression was associated with poorer overall survival [37]. For genes upregulated in PACSI-identified cells, several Reactome pathways related to cancer prognosis were significantly enriched, including recognition of DNA damage by PCNA-containing replication complex and PCNA-dependent long patch base excision repair [38–40] (Fig. 4G and Additional file 3).

To demonstrate that PACSI can provide novel biological insights, we computed the ssGSEA scores of the prognostic signature derived from upregulated genes in the PACSI-identified cells using three external bulk gene expression data and then stratified BRCA patients into high- and low-risk groups based on the lower quartile of PACSI-derived signature scores (Additional file 3). The results showed that PACSI-derived prognostic signature was robust across diverse independent datasets (Fig. 4H).

### Detection of cells related to immunotherapy response in melanoma

**Fig. 5:**
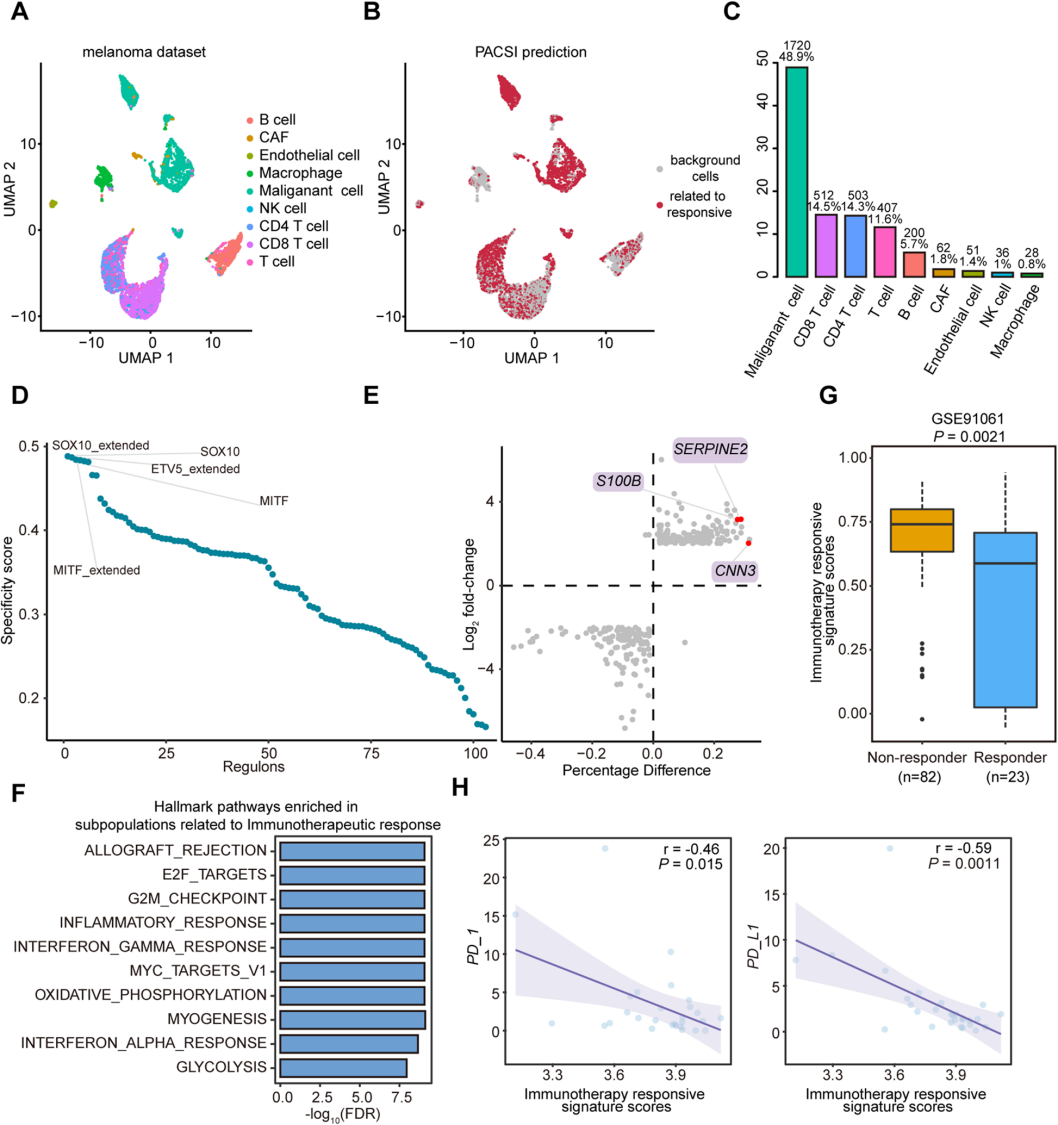
Application of PACSI on melanoma data. **A** The UMAP visualization of the melanoma scRNA-seq dataset. **B** The UMAP visualization of PACSI-identified cells. The red dots are PACSI-identified cells associated with immunotherapy response. **C** The distribution of PACSI-identified cells by cell types. **D** Rank for regulons in PACSI-identified cells associated with immunotherapy response based on RSS. **E** Differential gene expression analysis. The *x*-axis shows the difference in the percentage of cells expressing the gene between PACSI-identified cells and the others; the *y*-axis represents the log_2_ fold-change. **F** The top ten enriched Hallmark pathways in the PACSI-identified cells compared to other cells using GSEA. **G** Box plot shows the enrichment scores of the immunotherapy response signature in the responder and non-responder samples from the independent validation dataset. Two-tailed *P* value was calculated by Wilcoxon rank-sum test. **H** Scatterplots of immunotherapy responsive signature scores versus PD-1 and PD-L1 gene expression in bulk gene expression data. Pearson coefficient (*r*) and associated *P* value are reported

Immunotherapy is revolutionizing the treatment of cancer by enabling long-term tumor control [41]. To explore why some patients respond to immunotherapy and others do not, we performed PACSI analysis on a bulk mRNA expression profile of melanoma with clinical response information and a scRNA-seq matrix of 6,879 cells from 31 melanoma tumors [42, 43] (Fig. 5A). By performing PACSI, we identified 3519 cells that were associated with responder patients (Fig. 5B). As anticipated, malignant cells were the predominant cell type, accounting for 48.9% of total PACSI-selected cells, followed by CD8 T cells and CD4 T cells (Fig. 5C). These cell types are well known to be strongly correlated with immunotherapy [44, 45]. These results strongly suggested that PACSI can accurately identify cell subpopulations associated with immunotherapy response.

The top three regulators are shown in Fig. 5D, of which SOX10 had the highest RSS and was illustrated that could regulate ICI gene expression and anti-tumor immunity in melanoma [46] (Additional file 4). We also found a total of 284 DEGs between PACSI-identified cells and the others, among which 153 genes were upregulated and 131 genes were downregulated (Fig. 5E and Additional file 4). Notably, several upregulated genes have been demonstrated to be related to immunotherapy. For example, the expression level of *SERPINE2* was positively correlated with the level of CD4 T cells infiltration in the tumor, and it is well-known that CD4 T cells can enhance antitumor activity of cytotoxic T lymphocytes [44, 47] (Additional file 1: Supplementary Fig. 3a). Serum *S100B* levels have been reported to monitor response to immunotherapy in metastatic melanoma [48] (Additional file 1: Supplementary Fig. 3b). Moreover, the expression of *CNN3* is associated with the activity of several immune-related pathways and the expression of immune checkpoint molecules [49] (Additional file 1: Supplementary Fig. 3c). In addition, functional enrichment analysis revealed that the PACSI-identified cells associated with good response to immunotherapy had higher activity of immune-related pathways, including interferon-gamma (IFN-γ) response and interferon-alpha (IFN-α) response (Fig. 5F and Additional file 4). IFN-γ was found to drive clinical response to immune checkpoint blockade therapy in melanoma [50], suggesting that these PACSI-identified cells may improve the response to immunotherapy by regulating the IFN-γ response pathway. PACSI-derived signature scores associated with immunotherapy response were significantly different in non-responders and responders (Fig. 5G and Additional file 4).

Previous studies have found that Immune checkpoint inhibitors (ICI) were efficacious targets for anti-cancer immunotherapy [51, 52]. To further investigate the complex crosstalk between the PACSI-derived signature associated with good immunotherapy response and ICI genes (PD-1, PD-L1 and CTLA-4), we performed Pearson’s correlation analysis on the bulk dataset. As shown in Fig. 5H, the expression levels of PD-1 and PD-L1 were negatively correlated with the signature scores derived from PACSI-identified cells, suggesting that PACSI-identified cells may improve the response of patients to immunotherapy by upregulating the expression of ICI genes.

### Identification of cell subpopulations associated with COVID-19 disease

**Fig. 6:**
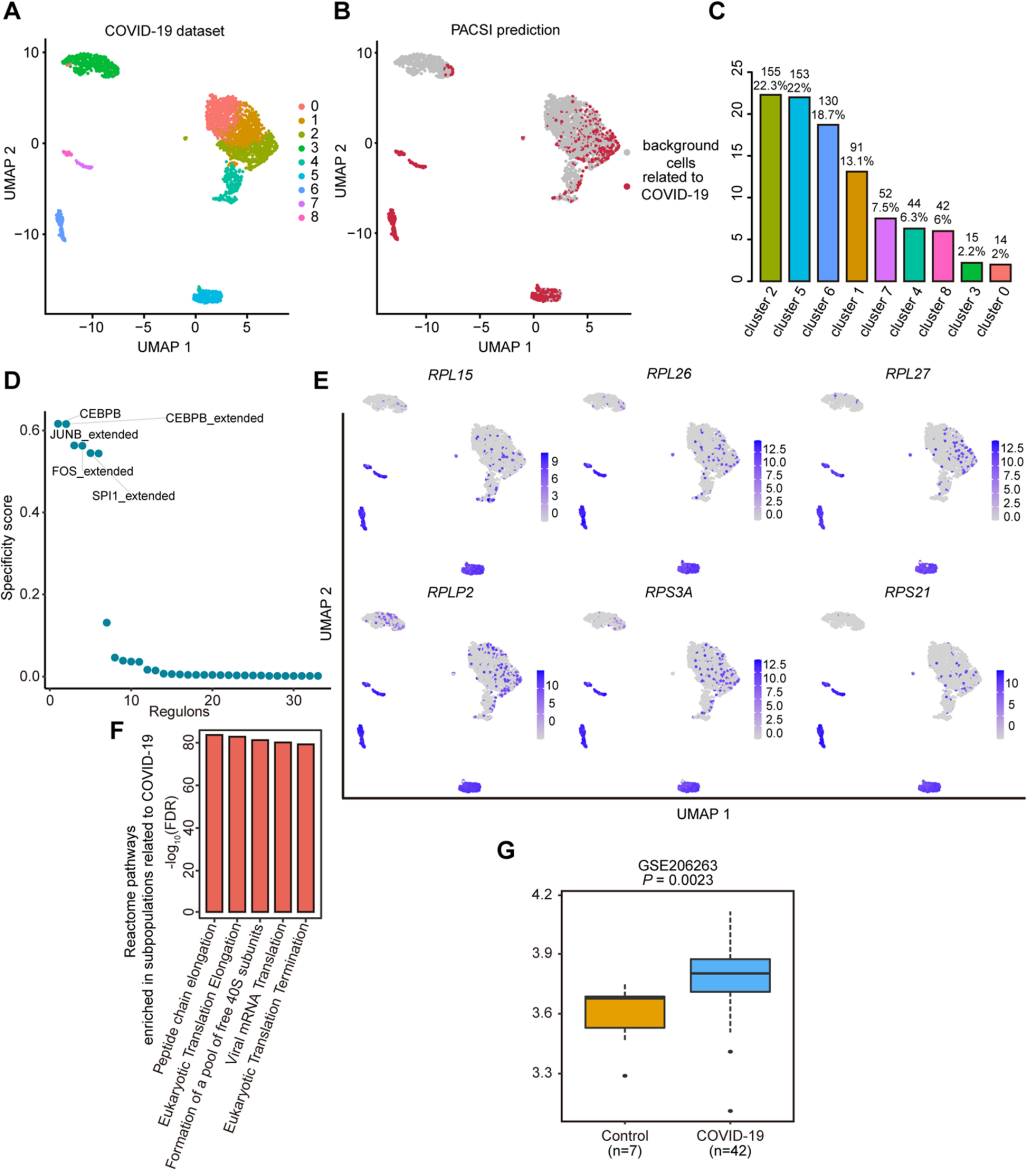
Application of PACSI on COVID-19 data. **A** The UMAP visualization of the COVID-19 scRNA-seq dataset. **B** The UMAP visualization of PACSI-identified cells. The red dots are PACSI-identified cells associated with COVID-19. **C** The distribution of PACSI-identified cells by cell clusters. **D** Rank for regulons in PACSI-identified cells associated with COVID-19 based on RSS. **E** The expression of vital genes in the single-cell data. **F** The top five Reactome pathways enrichment of genes that were expressed higher in the PACSI-identified cells. **G** Box plots show the enrichment scores of the COVID-19 signature in the COVID-19 and normal samples from the independent validation dataset. A two-sided Wilcoxon rank-sum test was performed to estimate the significance level

Beyond its utility in oncology, PACSI was also shown to offer insights into other diseases, such as COVID-19, which has been a global public health challenge in the past years. In this case study, we used a single-cell expression dataset of 2613 cells from the blood of a severe COVID-19 patient and a bulk gene expression matrix consisting of both COVID-19 and control samples to identify cells associated with COVID-19 [53] (Fig. 6A). Six hundred ninety-six cells were selected by PACSI to be related to COVID-19 disease, mainly from clusters 2 and 5 (Fig. 6B, C). Transcriptional regulatory analysis revealed that CEBPB, JUNB, FOS, and SPL1 were the most specific regulators for cells identified by PACSI (Fig. 6D and Additional file 5). Huang et al. have found that the expression of FOS was upregulated in patients and down-regulated in cured patients [54]. Moreover, after the virus reaches the blood immune cells, FOS and JUNB generated a wide range of antiviral responses by activating the expression of downstream effectors of the MAPK pathway [54]. In addition, FOS was found to have potential as a new target for puerarin in the treatment of COVID-19 [55].

Comparing COVID-19-associated cells with other cells, 930 genes altered significantly and most were overexpressed (Additional file 1: Supplementary Fig. 4 and Additional file 5), suggesting inductive events operating in the COVID-19 disease. We defined the percentage difference for a gene as the difference in the percentage of cells expressing that gene comparing PACIS-identified cells versus other cells. Intriguingly, the six genes with the largest percentage difference (*RPL15*, *RPL26*, *RPL27*, *PRLP2*, *RPS3A*, and *PRS21*) encode several ribosomal proteins that are components of the 60S and 40S subunits (Fig. 6E). Several studies have shown that nonstructural proteins of SARS-CoV-2 bind human ribosomal subunits to inhibit nonspecific immunity [56, 57]. Reactome pathway analysis also confirmed that the upregulated genes were significantly enriched in virus-associated pathways (e.g., viral mRNA translation) and translation-related pathways (e.g., peptide chain elongation, eukaryotic translation elongation, and eukaryotic translation termination) (Fig. 6F and Additional file 5). Bankar et al. [58] have found that mRNA translation pathways were altered significantly in response to COVID-19 infection, suggesting translation-related pathways may serve as potential targets for COVID-19 therapy.

To demonstrate that whether the PACSI-derived signature can distinguish samples from the COVID-19 disease from the normal tissue, we built a COVID-19 signature using the upregulated genes in cells identified by PACSI (Additional file 5). The result found that the COVID-19 signature was significantly different between disease and normal samples in the independent COVID-19 dataset (Fig. 6G).

### Application of PACSI on spatial transcriptomic data

**Fig. 7:**
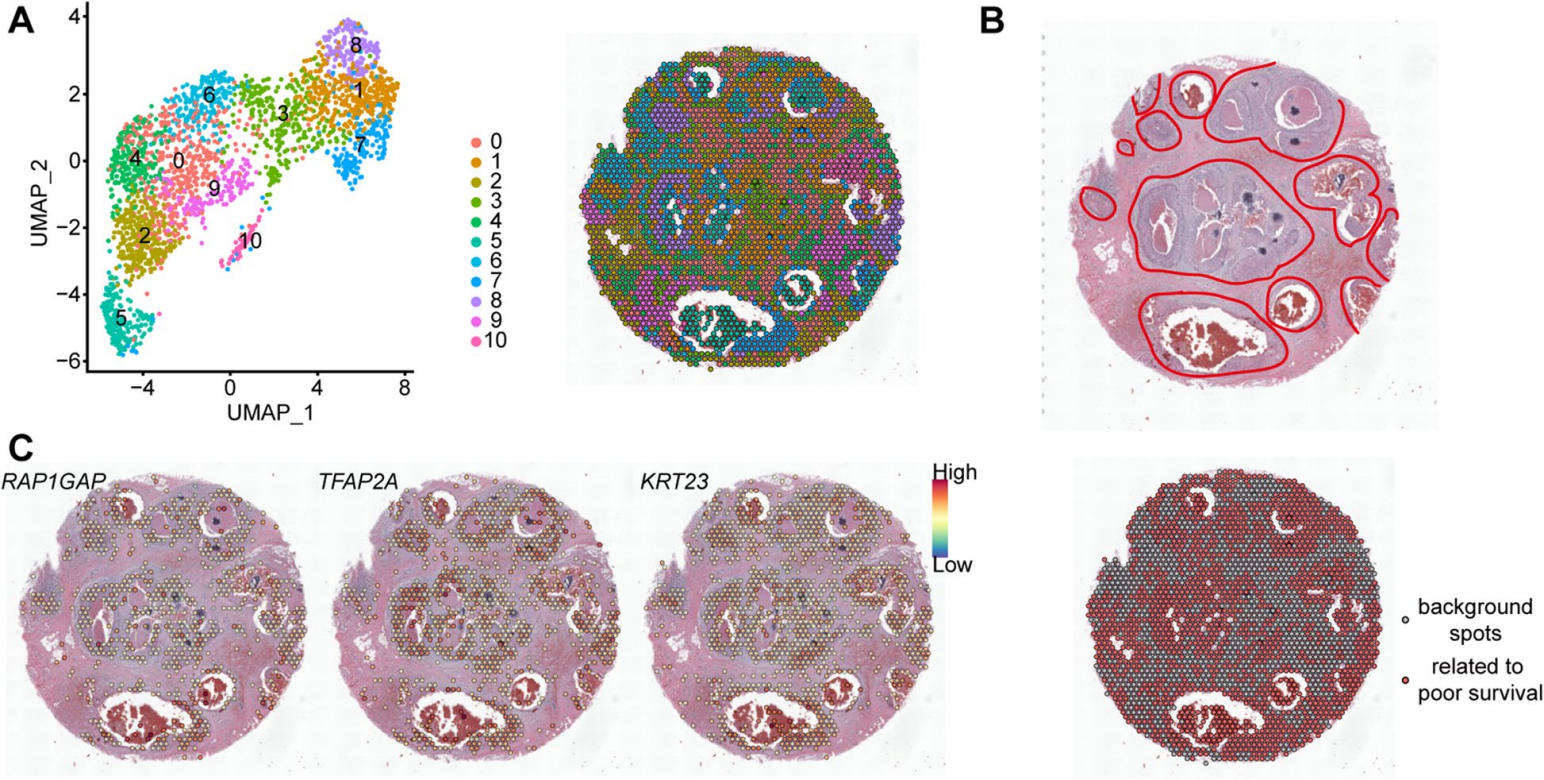
Application of PACSI on spatial transcriptomic data. **A** The spatial transcriptomic data was embedded in UMAP space (left) and unbiased clustering of spatial transcriptomics spots (right). **B** Histopathological annotations of human breast cancer sample in which malignant cells are highlighted in red circles (top). H&E images for spatial transcriptomic data overlaid with the locations of the spatial transcriptomics spots colored according to their annotation (bottom). The red dots are PACSI-identified spots associated with poor survival and the gray dots represent the rest of the spots in the spatial transcriptomic data. **C** Visualization of *RAP1GAP*, *TFAP2A*, and *KRT23* expression in spots under the tissue

To understand the spatial distribution of cell subpopulations associated with disease phenotypes, we performed PACSI on the Visium spatial gene expression data of breast ductal carcinoma and the TCGA-BRCA bulk dataset with survival information to identify spot subsets that were related to poor survival. The filtered spatial expression data contained 2518 spots which were separated in 11 clusters (Fig. 7A), and most spots that were associated with poor survival were located in overlapping anatomical locations with malignant cells [59] (Fig. 7B). Subsequently, we identified RAP1GAP, *TFAP2A*, KRT23, etc., as the highly upregulated genes (FDR < 0.01 and log_2_ fold-change > 1) of PACSI-identified spots (Additional file 6). Spots not identified by PACSI exhibited low expression or no expression of these genes (Fig. 7C). These upregulated genes were also found to be related to the BRCA progression [60–62]. It is worth nothing that *TFAP2A* was also found in the second real case (Fig. 4E), which suggested that *TFAP2A* may play a crucial role in the poor prognosis of patients with breast carcinoma. Functional annotation of these upregulated genes showed the strong enrichment of genes associated with cell-cell junctions and keratinization (Additional file 1: Supplementary Fig. 5 and Additional file 6). Cell-cell junctions played an important role in regulating cell proliferation and tumor cell migration [63]. Keratinization has been recognized as prognostic factors in many types of epithelial tumors [64]. These results provided a proof-of-concept for PACSI could infer the spatial locations of phenotype-related cells.

## Discussion

A major difficulty with single-cell data analysis is to infer the latent relationships between cell populations and disease phenotypes of interest. In order to overcome this challenge, we here proposed the PACSI algorithm, which integrates scRNA-seq and bulk expression data to identify cell subpopulations associated with disease phenotypes. In PACSI, network-based proximity was used to define similarity between cells and the phenotype of interest. Our hypothesis is that if a cell module is proximal to the disease phenotype of interest, it is more possible to be relevant than a distant cell in the network. PACSI was applied in simulated datasets with known ground-truth, as well as real HNSC, BRCA, melanoma, and COVID-19 disease datasets, to identify cells that are associated with disease phenotypes (e.g., disease vs. normal, poor survival vs. good survival and responder vs. non-responder). In addition, we also performed PACSI on spatial transcriptomics data of breast carcinoma, and the identified spots that were related to poor survival were validated by pathology annotation. The results based on these validation datasets showed that PACSI was a generally applicable tool in a wide variety of disease phenotypes data.

One important hyper-parameter in PACSI is the size of cell/sample signatures, which determines the size of cell/sample modules in the network when calculating the proximity between cells and the phenotype of interest. If the size of signatures is too small, gene signatures may not reflect the transcriptional characteristics of cells or samples. In contrast, if the size of signatures is too large, too much noise may be included. We applied PACSI under a wide range of the sizes of signatures using a simulated dataset and found that PACSI performed best in simulated data when the gene signature size is 150-gene. However, a proper size depends on the size and topological structure of data, which may vary from study to study. The input network data can either be provided by the user or be constructed directly by PACSI. When no PPI network data is available, a co-expression network calculated by PACSI will be used instead of PPI networks.

Due to the high dropout rates of scRNA-seq data, we have explored the effect of missing value imputation on the performance of PACSI. We applied MAGIC [65] to impute the gene expression for the simulated single-cell data. We found that imputation had little impact on the overall performance of PACSI, which suggested that PACSI was robust against dropout noise in single-cell RNA-seq data (Additional file 1: Supplementary Fig. 6). Since the PACSI algorithm only focuses on the phenotype of interest (phenotype-1) and the opposite phenotype (phenotype-0) is not covered in the method, we compared the differences in the association between the two phenotypes and the cells identified by the PACSI method. The results showed that PACSI-identified cells exhibit significantly lower correlation *P* values with phenotype 1 compared to phenotype 0 in all four single-cell data cases, which demonstrates the accuracy and robustness of the method (Additional file 1: Supplementary Fig. 7). We also measured the total run time and memory requirements for each real case, as well as the total time and memory consumption of PACSI as the number of cells increased. Our findings showed that they were within an acceptable range (Additional file 1: Supplementary Table 1 and Additional file 1: Supplementary Table 2).

The greatest advantage of PACSI is the integration of biological and topological information to guide the identification of phenotype-specific cells. In addition, it is very difficult to select the number of clusters for various datasets, and PACSI does not require any unsupervised clustering. We compared the performance of PACSI with two existing algorithms (Scissor and DEGAS) on a simulated dataset and observed that PACSI outperformed the compared methods. We also demonstrated that PACSI could be applied on HNSC, BRCA, melanoma, COVID-19, and spatial transcriptomic datasets, which suggested that PACSI could be generalized to diverse tasks.

A potential drawback of PACSI is that the directionality of action of the identified cells on the phenotype of interest cannot be determined. Whether these phenotype-related cells identified by PACSI promote or inhibit the changes in phenotype of patients is the main target of our next study. Furthermore, due to the incompleteness of the current networks, the performance of our methods can be improved as more information becomes available.

## Conclusions

In summary, our results suggest that network-based cell-phenotype proximity offered an unbiased measure of the relationship between the cells and disease phenotypes of interest and could be a powerful and effective solution to identify cell subpopulations associated with disease phenotype. As scRNA-seq technology matures and single-cell datasets grow rapidly, we believe that PACSI will assist in unraveling the underlying biological mechanisms behind complex patient diseases and developing novel cell-targeted therapeutic strategies.

## Methods

### PACSI workflow

#### Input data

PACSI requires a single-cell expression matrix, a bulk expression matrix, phenotype labels, and a PPI network as input. The two expression matrices should be TPM/FPKM-normalized with rows corresponding to genes and columns corresponding to cells/samples. PACSI first performs sample-wise *z*-score normalization for the bulk expression matrix and then uses Seurat to scale the single-cell data and identify high variance genes. The phenotype labels $y$ matched with the bulk dataset should be binary groups (1: the phenotype of interest; 0: the control phenotype). For example, $y=\left[1, 0, 1, 0\right]$ indicates the phenotype of the first and third samples in the bulk matrix are of interest while the phenotype of the second and fourth samples are the control. The network data can either be provided by the user or be constructed directly by PACSI. If the PPI network is not available, PACSI will construct co-expression networks instead. PACSI defines the similarity co-expression matrix based on the significance ${S}_{ij}$ of Pearson’s correlation between the $i$ th gene and the $j$ th gene, then the similarity matrix calculated for all pairwise genes in the single-cell dataset is transformed into a binary network adjacency matrix $\mathbf{A}$ using the following function:

1 $${\mathbf{A}}_{ij}=\left\{\begin{array}{cc}1& if\;{S}_{ij}<0.05 \\ 0& otherwise\end{array}\right.$$

#### Computation of cell signatures and sample signatures

To obtain the gene signature for each bulk sample and each cell, the scRNA-seq matrix and bulk gene expression matrix are first transformed into two rank-based matrices separately. Let $m$ and $n$ denote the total number of cells and genes in the single-cell data, respectively. A rank-based single-cell expression matrix $\mathbf{C}={\left({c}_{ij}\right)}_{n\times m}$ is first constructed, where ${{\varvec{c}}}_{ij}$ is the rank of the expression value of gene $i$ in cell $j$ compared with all other cells in the dataset divided by the number of cells. The rank information represents the relative abundance of genes in a cell relative to all other cells in the dataset. The 150 genes with the highest relative abundance for cell $j$ are identified as the cell $j$ signature. The gene signature of each sample in the bulk data is calculated using the same method. After that, for each gene in the signature, PACSI maps the gene symbol to UniProt ID using the R package clusterProfiler [66]. The UniProt IDs are used to map cell/sample signatures to the corresponding proteins in the PPI network. This indicates that each cell/sample signature induces a network module. To calculate the path lengths between cell modules and sample modules at the next step, the largest connected component of the PPI network is extracted using the igraph package [67].

#### Correlation scores between cells and the phenotype of interest

To calculate the proximity of cells for the phenotype of interest, PACSI first employs the following distance measure to compute the path lengths between cell modules and sample modules.

2 $$d\left(S,C\right)=\frac{1}{\Vert C\Vert }\sum_{c\epsilon C}{min}_{s\epsilon S}d\left(s,c\right)$$

where $S$ is the set of proteins in the sample modules and $C$ is the set of proteins in the cell modules. $d\left(s,c\right)$ is the shortest path length between nodes $s$ and $c$ in the network. Given $P$, the sample set of the phenotype of interest, we define the distance between the cell module and the phenotype of interest as follows:

3 $$d\left(P,C\right)=\frac{1}{\Vert P\Vert }\sum_{S\epsilon P}d(S,C)$$

#### Significance test

To evaluate the statistical significance of the distance between a cell module and the phenotype of interest, the first step is to create a background distance distribution corresponding to the actual distance by selecting randomly a set of proteins matching the size of the original cell module in the network. The background distance distribution is created by computing the proximity between the random cell module and the phenotype of interest, a procedure repeated many, e.g., 100 times. Lastly, the empirical *P* value is defined as the number of random distances with lower distance scores than the actual distance score divided by the overall number of random cell modules. The empirical *P* value lower than 0.05 is considered significant.

### Simulated datasets setup

To test the performance of PACSI, the simulated single-cell data with 5000 cells and 10,000 genes are generated using Splatter [16]. These cells are from 10 cell clusters with a group probability of 0.1, and the probability of each gene being expressed differently is also 0.1. We use the splatSimulate function to simulate scRNA-seq count data, utilizing the default parameters of the function except those specifically mentioned above. The raw count data is converted to a TPM matrix using the calculateTPM function from the scuttle R package. Then, we split these 5000 cells into two parts, with cells in cluster 1 assigned to be associated with the phenotype of interest, while the other cells are assigned as controls. We generated simulated bulk expression data for 500 tissue samples, consisting of 250 samples labeled 1 with an interesting phenotype, and another 250 samples labeled 0 representing the control phenotype. The gene expression values of each bulk tissue sample labeled 1 are generated by randomly selecting 100 cells with replacement from cluster 1 of the simulated single-cell data and averaging the expression values of these 100 cells. Similarly, the gene expression values of each bulk sample labeled 0 are obtained by averaging the expression of 100 cells randomly selected with replacement from clusters other than cluster1.

### Datasets and pre-processing

#### HNSC scRNA-seq data

The HNSC single-cell dataset used in this study was downloaded from the Gene Expression Omnibus (GEO: accession number: GSE103322) [68, 69]. This scRNA-seq data contains 5902 single cells from 18 patients with oral cavity tumors. We removed cells from lymph nodes and cells whose cell type could not be identified and focused our analysis on the remaining 4244 cells. All 4244 cells containing 1427 cancer cells and 2817 non-cancer cells were used to identify cell subsets that were associated with HNSC.

#### HNSC bulk data

The TCGA-HNSC bulk data and phenotype information were downloaded using the GDCRNATools R package [70]. Read counts per gene were further converted into transcript per million (TPM) quantification and log_2_-transformed [log_2_ (TPM+1)]. In TCGA-HNSC, there are in total of 522 tumors, 44 normal samples, and 2 metastatic samples. After removing two metastatic samples, the remaining 566 samples were used as the input of PACSI.

#### HNSC validation data

The independent HNSC dataset (accession numbers: GSE143083) was downloaded from the GEO database [71, 72]. The Ensemble gene ids were mapped to gene symbols using clusterProfiler [66]. We removed genes that were not detected in 50% of the samples.

#### BRCA scRNA-seq data

The single-cell dataset of 1534 cells from six triple-negative breast cancer tumors was downloaded from the GEO (accession number: GSE118389) [27, 73]. The expression matrix was then log_2_-transformed.

#### BRCA bulk data

The breast carcinoma bulk fragments per kilobase of transcript per million fragments mapped (FPKM) gene expression data and survival information were downloaded from UCSC Xena [28]. When mapping the Ensemble ids to gene symbols, the mean expression values of Ensemble ids mapped to the same gene were used. We considered patients who survived past 3 years (regardless of status) as good survivals and patients that deceased in less than 3 years as poor survivals. Living patients with a survival time of fewer than 3 years were excluded from this study. Finally, we obtained 72 poor survival samples and 435 good survival samples.

#### BRCA validation data

The three external bulk BRCA microarray expression data were downloaded from the GEO (accession number: GSE1456, accession number: GSE4922, accession number: GSE25066) [74–76] with GEOquery [77] to test the efficacy of the prognostic signature. The GSE1456 dataset contains gene expression data collected from 159 tumor tissues of breast cancer patients with overall survival information [78]. The GSE4922 dataset consists of gene expression profiles of 347 primary invasive breast tumors with disease-free survival information, analyzed using Affymetrix microarrays [79]. GSE25066 is a microarray-based gene expression dataset comprising 508 breast cancer samples, each with recurrence-free survival information included [80].

#### Melanoma scRNA-seq data

The scRNA-seq data of 7186 cells from 31 melanoma tumors was downloaded from GEO (GSE115978) [42, 81]. In our initial data inspection, 307 cells with no defined cell type were removed. We used the remaining 6879 cells as the input of PACSI.

#### Melanoma bulk data

Twenty-three melanoma patients with known clinical response information were collected from the GEO (accession number: GSE78220) [43, 82]. This dataset includes 13 non-responders and 10 responders.

#### Melanoma validation data

The independent dataset was downloaded from the GEO (accession number: GSE91061) [83, 84]. The Entrez IDs were mapped to gene symbols using clusterProfiler. We removed genes that were not detected in 50% of the samples.

#### COVID-19 scRNA-seq data

The single-cell expression data was obtained from the GEO (accession number: GSE157344) [53, 85]. In order to reduce the size of the dataset to increase the speed of PACSI operation, we only kept 2613 cells from a severe COVID-19 sample (accession number: GSM4762161) as the scRNA-seq input of PACSI.

#### COVID-19 bulk data

The COVID-19 bulk gene expression matrix was downloaded from GEO (accession number: GSE196822) [86, 87]. We removed 6 patients with viral–bacterial co-infections and focused our analysis on the remaining 34 COVID-19 samples and 9 healthy samples.

#### COVID-19 validation data

The independent dataset was downloaded from the GEO (accession number: GSE206263) [88, 89] to test whether the COVID-19 signature is effective in distinguishing COVID-19 patients from normal controls. GSE206263 includes 42 COVID-19 samples and 7 healthy samples. Read counts per gene were further converted into transcript per million (TPM) quantification using IOBR [90] and then log_2_-transformed.

#### Spatial transcriptomic data

The spatial transcriptomic dataset of breast carcinoma was retrieved from the 10x website (https://www.10xgenomics.com/resources/datasets). Read counts per gene were first converted into transcripts per million (TPM) quantification using the IOBR package. Then, the main preprocessing analysis was performed using the Seurat package. The dataset was normalized by variance stabilizing transformation using the SCTransform function. Spot clusters were generated through dimensionality reduction and clustering.

#### PPI data

We first downloaded 69,567 experimentally verified human PPIs data from the MINT database [18] and used this data for all real cases in this study. Uniprot IDs were used to map genes in cell/sample signatures to the corresponding proteins in the interactome.

### Gene regulatory network analysis

Here, we apply SCENIC [91] to explore the gene regulatory networks of cell subpopulations identified by PACSI through four steps: (1) single-cell datasets are used to mine co-expression modules between transcription factors (TFs) and their potential target genes; (2) to prune co-expression modules, TFs and their direct targets are inferred by R package RcisTarget [91]. RcisTarget can identify transcription factors that are significantly enriched in the target genes using a database that contains genome-wide rankings for each motif; (3) the regulon activity score (RAS) is calculated to quantify the activity of regulons in each cell. Each regulon represents a TF along with its direct target genes; (4) for each cell subpopulation, the key regulons with high RSSs are predicted by an entropy-based strategy. RSS represents the activity of regulons in cell subpopulations.

### Differential expression and enrichment analysis

The differentially expressed genes are computed using the Wilcoxon rank-sum test as applied in the FindMarkers function in Seurat [92]. DEGs are obtained using a minimum absolute log_2_ fold-change of 2 and a maximum Bonferroni adjusted *P* value of 0.01. After that, the Hallmark gene sets (h.all.v7.5.1.symbols.gmt) downloaded from the Molecular Signatures Database (MSigDB) are used to perform gene set enrichment analyses (GSEA) using the clusterProfiler package [66]. The clusterProfiler package supports enrichment analysis with GSEA and adjusts *P* values for multiple hypothesis testing. Reactome pathway enrichment analyses are performed using the hypergeometric test as implemented in ReactomePA [93].

### Computation of signature enrichment scores

To demonstrate the characteristic of cells identified by PACSI, we compare PACSI-derived signature scores between groups of samples with distinct phenotypes in the independent dataset. We define the genes significantly upregulated in PACSI-identified cells relative to other cells as PACSI-derived gene signatures (log_2_ fold-change > 2 and FDR < 0.01). And then, the ssGSEA method implemented in GSVA [94] is used to calculate the signature enrichment score for each sample. Specifically, we use the gsva function in the GSVA package, with the original Kuiper statistic parameter and default Gaussian kernel parameters. Other parameters are set to their default values. The Wilcoxon rank-sum test is performed to examine the differences of the PACSI-derived signature enrichment scores between samples with distinct phenotypes.

### Survival analysis

To explore the association between the prognostic cells identified by PACSI and survival risks, we construct a prognostic signature by selecting the upregulated genes in cells identified by PACSI. The signature score is generated for each sample using the ssGSEA method and then the lower quartile of signature scores is defined as the cutoff value to separate samples into high-risk group and low-risk group. We perform the Kaplan-Meier analysis to visualize the survival distributions of two groups and use the log-rank test to assess the difference between two survival distributions. Specifically, we use the survfit function from the survival package to calculate Kaplan–Meier survival estimate and the ggsurvplot function from the survminer package to plot the survival curve.

### Statistical analysis

All statistical analyses are conducted in R (version 4.1.1). The Wilcoxon rank-sum test is used to identify DEGs. For Hallmark pathway enrichment analysis, *P* values are calculated by permutation test. For Reactome pathway enrichment analysis, the hypergeometric test is used. We use the Wilcoxon rank-sum test to compare the signature scores between groups of samples with distinct phenotypes. The correlations of immune-related genes and signatures identified by PACSI are conducted using Pearson correlation by the stats package. The log-rank test is used to compare the difference between survival curves. Benjamini-Hochberg FDR method is used to adjust *P* values for multiple tests [95]. If the FDR is lower than 0.01, it is reported as statistically significant.

## Supplementary Information

**Additional file 1:** **Supplementary Fig 1.** A, The UMAP visualization of simulated single-cell data. B, The distribution of PACSI-identified cells by cell clusters. C, The PR curves of three methods on the simulated dataset. **Supplementary Fig 2.** Violin plots show the expression level of the CDH3 gene in PACSI-identified cells (*n* = 46) and all the other cells (*n* = 4198). **Supplementary Fig 3.** The expression of vital genes in the melanoma single-cell data. **Supplementary Fig 4.** Differential gene expression analysis. The x-axis shows the difference in the percentage of cells expressing the gene between PACSI-identified cells associated with COVID-19 and the others, the y-axis represents the log_2_ fold-change. **Supplementary Fig 5.** The top five Reactome enrichment of genes that were expressed higher in the PACSI-identified spots, ordered by -log_10_(FDR). **Supplementary Fig 6.** The ROC curves of PACSI on simulated data with and without dropout. **Supplementary Fig 7.** Box plots of HNSC case (A), BRCA case (B), melanoma case (C) and COVID-19 case (D) show the *P* values between PACSI-identified cells and the disease phenotype of interest or control phenotype. **Supplementary Table 1.** The run time and memory requirements of PACSI on real cases. **Supplementary Table 2.** The run time and memory requirements of PACSI for different numbers of cells.

**Additional file 2.** Table with differential expression genes in PACSI-identified cells associated with HNSC versus all other cells. Table with Hallmark pathways enrichment of differential expression genes between PACSI-identified cells associated with HNSC versus all other cells. Table with full list of genes in HNSC signature. Table with the specificity scores of regulons in PACSI-identified cells associated with HNSC.

**Additional file 3.** Table with differential expression genes in PACSI-identified cells associated with poor survival versus all other cells. Table with the Reactome pathways enrichment of upregulated genes in PACSI-identified cells associated with poor survival. Table with full list of genes in prognostic signature. Table with the specificity scores of regulons in PACSI-identified cells associated with poor survival.

**Additional file 4.** Table with differential expression genes in PACSI-identified cells associated with good immunotherapy response versus all other cells. Table with Hallmark pathways enrichment of differential expression genes between PACSI-identified cells associated with good immunotherapy response versus all other cells. Table with full list of genes in PACSI-derived signature associated with good immunotherapy response. Table with the specificity scores of regulons in PACSI-identified cells associated with good immunotherapy response.

**Additional file 5.** Table with differential expression genes in PACSI-identified cells associated with COVID-19 versus all other cells. Table with the Reactome pathways enrichment of upregulated genes in PACSI-identified cells associated with COVID-19. Table with full list of genes in COVID-19 signature. Table with the specificity scores of regulons in PACSI-identified cells associated with COVID-19.

**Additional file 6.** Table with differential expression genes in PACSI-identified spots associated with poor survival versus all other spots. Table with the Reactome pathways enrichment of upregulated genes in PACSI-identified spots associated with poor survival. Table with full list of the upregulated genes in PACSI-identified spots associated with poor survival versus all other spots.

## Data Availability

All data generated or analyzed during this study are included in this published article, its supplementary information files and publicly available repositories. We download the scRNA-seq data of HNSC, BRCA, melanoma, and COVID-19 from the NCBI Gene Expression Omnibus (GEO; https://www.ncbi.nlm.nih.gov/geo/) with the following accession numbers: HNSC scRNA-seq: GSE103322 (https://www.ncbi.nlm.nih.gov/geo/query/acc.cgi?acc=GSE103322) [68, 69]; BRCA scRNA-seq: GSE118389 (https://www.ncbi.nlm.nih.gov/geo/query/acc.cgi?acc=GSE118389) [27, 73]; melanoma scRNA-seq: GSE115978 (https://www.ncbi.nlm.nih.gov/geo/query/acc.cgi?acc=GSE115978) [42, 81]; COVID-19 scRNA-seq: GSE157344 (https://www.ncbi.nlm.nih.gov/geo/query/acc.cgi?acc=GSE157344) [53, 85]. The TCGA-HNSC bulk data and phenotype information are downloaded using the GDCRNATools R package [70]. The breast carcinoma bulk gene expression data and survival information are downloaded from UCSC Xena (http://xena.ucsc.edu/). We get the bulk data of melanoma and COVID-19 from GEO with the accession numbers GSE78220 (https://www.ncbi.nlm.nih.gov/geo/query/acc.cgi?acc=GSE78220) [43, 82] and GSE196822 (https://www.ncbi.nlm.nih.gov/geo/query/acc.cgi?acc=GSE196822) [86, 87], respectively. We download the validation data from GEO with the accession numbers: HNSC validation: GSE143083 (https://www.ncbi.nlm.nih.gov/geo/query/acc.cgi?acc=GSE143083) [71, 72]; BRCA validation: GSE1456 (https://www.ncbi.nlm.nih.gov/geo/query/acc.cgi?acc=GSE1456) [76, 78], GSE4922 (https://www.ncbi.nlm.nih.gov/geo/query/acc.cgi?acc=GSE4922) [74, 79], GSE25066 (https://www.ncbi.nlm.nih.gov/geo/query/acc.cgi?acc=GSE25066) [75, 80]; melanoma validation: GSE91061 (https://www.ncbi.nlm.nih.gov/geo/query/acc.cgi?acc=GSE91061) [83, 84]; COVID-19 validation: GSE206263 (https://www.ncbi.nlm.nih.gov/geo/query/acc.cgi?acc=GSE206263) [88, 89]. The spatial transcriptomic dataset of breast carcinoma is retrieved from the 10x website (https://www.10xgenomics.com/resources/datasets). We download 69,567 experimentally verified human PPIs data from the MINT database (http://www.ebi.ac.uk/Tools/webservices/psicquic/mint/webservices/current/search/query/species:human). The code for this project is integrated with an R package PACSI, which can be obtained on Github Repository (https://github.com/Chonghui-Liu/PACSI-project) and Zenodo (https://doi.org/10.5281/zenodo.8042616).

## Abbreviations

scRNA-seq: Single-cell RNA sequencing
PPI: Protein-protein interaction
AUC: Area under the receiver operating characteristic curve
ROC: Receiver operating characteristic
TF: Transcription factor
RAS: Regulon activity score
RSS: Regulon specificity score
DEG: Differentially-expressed gene
GSEA: Gene set enrichment analysis
ssGSEA: Single-sample gene set enrichment analysis
HNSC: Head and neck squamous cell carcinoma
OS: Overall survival
DFS: Disease-free survival
RFS: Recurrence-free survival
FDR: False discovery rate
PR: Precision-recall
AUPR: Area under the precision-recall curve
