# Supplementary material for: Identification of cell subpopulations associated with disease phenotypes from scRNA-seq data using PACSI
Source: BMC Biol. 2023 Jul 19;21:159. doi: 10.1186/s12915-023-01658-3 (PMC10354926; doi:10.1186/s12915-023-01658-3)
Supplement: Supplementary file 1 — Additional file 1: Supplementary Fig 1. A, The UMAP visualization of simulated single-cell data. B, The distribution of PACSI-identified cells by cell clusters. C, The PR curves of three methods on the simulated dataset. Supplementary Fig 2. Violin plots show the expression level of the CDH3 gene in PACSI-identified cells (n = 46) and all the other cells (n = 4198). Supplementary Fig 3. The expression of vital genes in the melanoma single-cell data. Supplementary Fig 4. Differential gene expression analysis. The x-axis shows the difference in the percentage of cells expressing the gene between PACSI-identified cells associated with COVID-19 and the others, the y-axis represents the log2 fold-change. Supplementary Fig 5. The top five Reactome enrichment of genes that were expressed higher in the PACSI-identified spots, ordered by -log10(FDR). Supplementary Fig 6. The ROC curves of PACSI on simulated data with and without dropout. Supplementary Fig 7. Box plots of HNSC case (A), BRCA case (B), melanoma case (C) and COVID-19 case (D) show the P values between PACSI-identified cells and the disease phenotype of interest or control phenotype. Supplementary Table 1. The run time and memory requirements of PACSI on real cases. Supplementary Table 2. The run time and memory requirements of PACSI for different numbers of cells. [file 12915_2023_1658_MOESM1_ESM.doc]

**Supplementary Materials:**

**Identification of cell subpopulations associated with disease phenotypes from scRNA-seq data using PACSI**

Chonghui Liu^1, 2^, Yan Zhang^3^, Xin Gao^4, 5^,*, and Guohua Wang^2, 6^,*

^1^ College of Life Science, Northeast Forestry University, Harbin 150040, China.

^2^ College of Information and Computer Engineering, Northeast Forestry University, Harbin 150040, China.

^3^ Department of Ophthalmology, the Second Affiliated Hospital of Harbin Medical University, Harbin 150086, China.

^4^ Computer Science Program, Computer, Electrical and Mathematical Sciences and Engineering (CEMSE) Division, King Abdullah University of Science and Technology (KAUST), Thuwal 23955-6900, Kingdom of Saudi Arabia.

^5^ KAUST Computational Bioscience Research Center (CBRC), King Abdullah University of Science and Technology, Thuwal 23955-6900, Kingdom of Saudi Arabia.

^6^ School of Computer Science and Technology, Harbin Institute of Technology, Harbin 150001, China.

***Corresponding authors**:

Xin Gao. E-mail: xin.gao@kaust.edu.sa. Tel: +966-12-8080323; Guohua Wang. E-mail: ghwang@nefu.edu.cn. Tel: +86 13946094199

**1. Supplementary Figures**

**
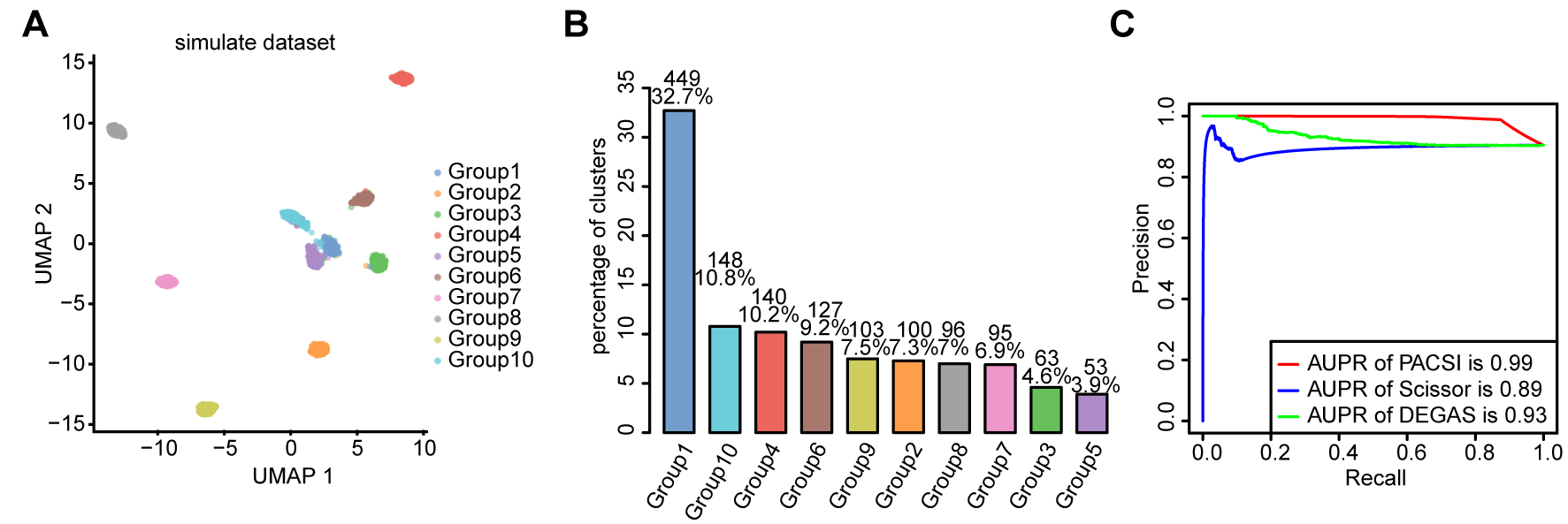
**

**Supplementary Fig. 1. a**, The UMAP visualization of simulated single-cell data. **b**, The distribution of PACSI-identified cells by cell clusters. **C**, The PR curves of three methods on the simulated dataset.


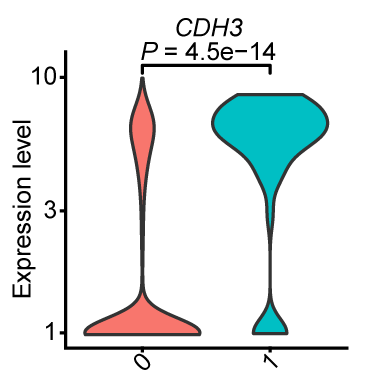


**Supplementary Fig. 2.** Violin plots show the expression level of the *CDH3* gene in PACSI-identified cells (n=46) and all the other cells (n=4198). The two-tailed *P* value was calculated by the Wilcoxon rank sum test.


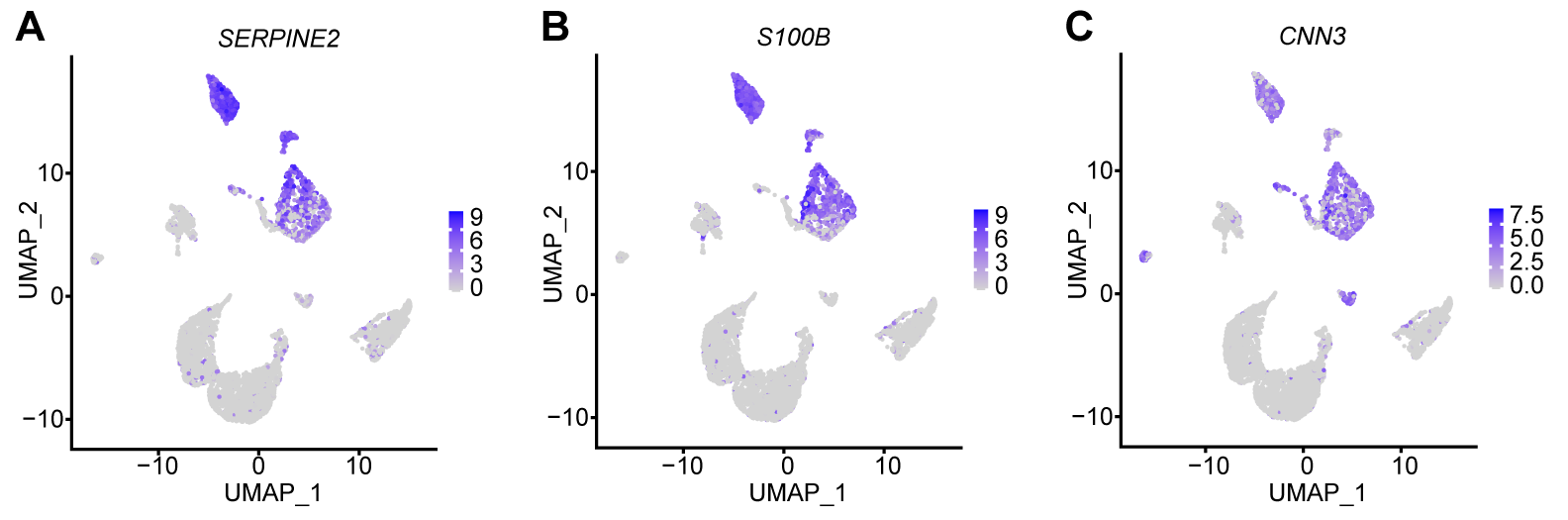


**Supplementary Fig. 3.** The expression of vital genes in the melanoma single-cell data.


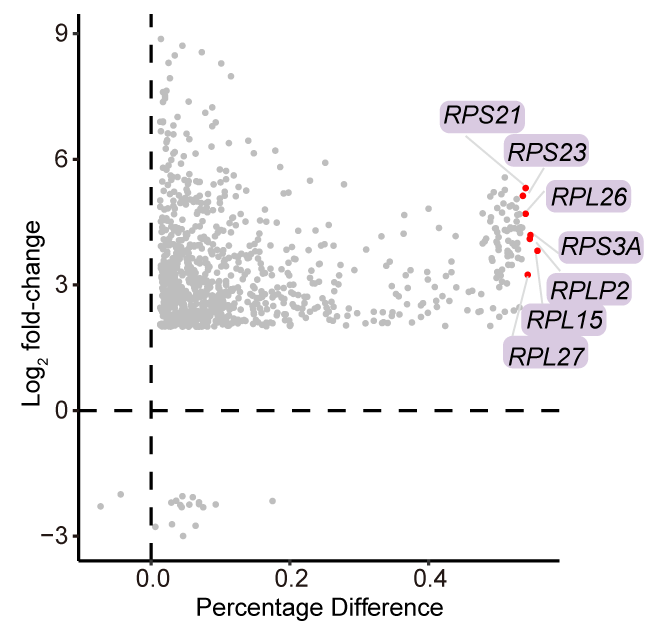


**Supplementary Fig. 4.** Differential gene expression analysis. The x-axis shows the difference in the percentage of cells expressing the gene between PACSI-identified cells associated with COVID-19 and the others, the y-axis represents the log_2_ fold-change.


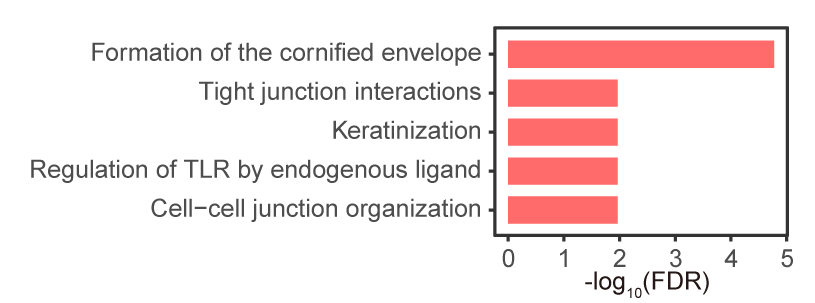


**Supplementary Fig. 5.** The top five Reactome enrichment of genes that were expressed higher in the PACSI-identified spots, ordered by -log_10_(FDR).


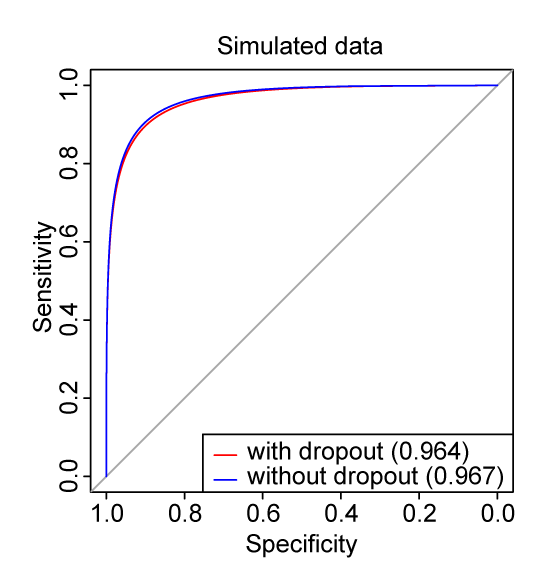


**Supplementary Fig. 6.** The ROC curves of PACSI on simulated data with and without dropout.


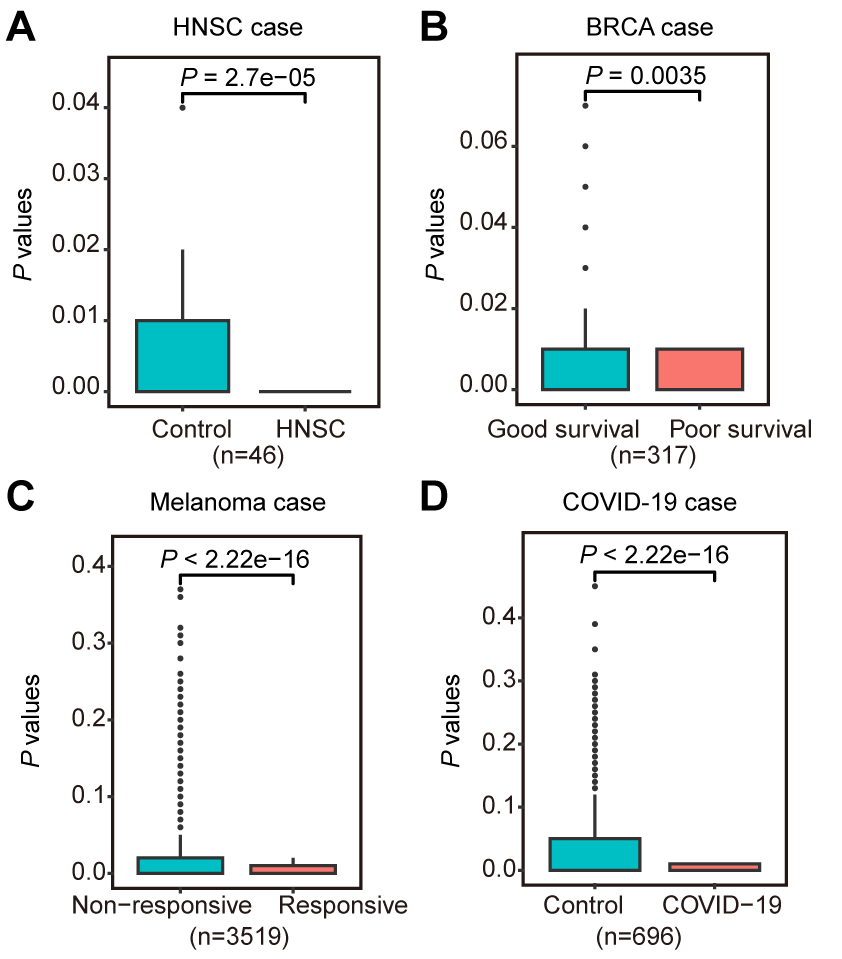


**Supplementary Fig. 7.** Box plots of HNSC case (A), BRCA case (B), melanoma case (C) and COVID-19 case (D) show the *P* values between PACSI-identified cells and the disease phenotype of interest or control phenotype. A two-sided Wilcoxon rank-sum test was performed to estimate the significance level.

**2. Running time and memory requirements of PACSI**

The run time and memory requirements of PACSI on real cases were provided in this section. These results were collected on a server with a 2.4 GHz Intel Xeon Platinum 8260 CPU and 5.9T memory. The run time and the memory requirements of PACSI on each real case were shown in Supplementary Table 1. To evaluate the time and memory consumption of PACSI as the number of cells increases, we regenerated simulated single-cell data ranging from 5,000 to 30,000 cells. The time and memory consumption of PACSI was shown in Supplementary Table 2.

**Supplementary Table 1.** The run time and memory requirements of PACSI on real cases.

| Experiments | #Cell | Running time  (unit: second) | Memory usages  (unit: Megabyte) |
| --- | --- | --- | --- |
| HNSC case | 4,244 | 1,606 | 1,628 |
| BRCA case | 1,534 | 1,258 | 989 |
| Melanoma case | 6,879 | 1,898 | 1894 |
| COVID-19 case | 2,613 | 1,399 | 806 |
| Spatial transcriptomic case | 2,518 (spots) | 1,470 | 1,638 |

**Supplementary Table 2.** The run time and memory requirements of PACSI for different numbers of cells.

| #Cell | Running time  (unit: second) | Memory usages  (unit: Megabyte) |
| --- | --- | --- |
| 5000 | 1,633 | 1,208 |
| 10,000 | 2,375 | 1,720 |
| 15,000 | 3,078 | 2,243 |
| 20,000 | 3,915 | 2,755 |
| 25,000 | 4,710 | 3,267 |
| 30,000 | 5,663 | 3,779 |
